# Objective Three-Dimensional Analysis of Cranial Morphology

**Published:** 2008-04-09

**Authors:** Jeffrey R Marcus, Leahthan F Domeshek, Rajesh Das, Sean Marshall, Roger Nightingale, Tracey H Stokes, Srinivasan Mukundan

**Affiliations:** Interdisciplinary Craniofacial Imaging Lab, Duke University, Durham, NC; Division of Plastic and Reconstructive Surgery, Duke University Medical Center, Durham, NC; Department of Radiology, Duke University Medical Center, Durham, NC; Department of Biomedical Engineering, Duke University, Durham, NC

## Abstract

**Objective:** The lack of adequate means to objectively characterize cranial shape contributes to ongoing controversies in the surgical management of craniosynostosis. Cranial shape analysis must address relevant clinical questions objectively and thoroughly and must be broadly applicable across the spectrum of normal and abnormal. Herein, we demonstrate and statistically validate an automated computed tomography (CT)-based application for 3-dimensional characterization of skull morphology. The technology is intended for application to diagnostic imaging, surgical planning, and outcomes assessment. **Methods:** Three-dimensional vector analysis (3DVA) was applied to craniofacial CT data, generating three-dimensional cranial surface point clouds. **Validation:** To assess accuracy, measurements derived from the 3DVA analysis of a CT scan of a skull phantom were compared to those made directly from the Digital Imaging and Communications in Medicine data on a Vitrea workstation. To assess reproducibility, 3 readers independently analyzed human head CT scans using 3DVA. **Application:** A normative database of 86 age-incremental pediatric patients was created. Preoperative craniosynostosis case datasets were analyzed using 3DVA and were compared with age-matched normative datasets. **Results:** Accuracy and reproducibility of less than 1% mean error and less than 0.5 mm standard error in all cases validated 3DVA-derived distances. Three-dimensional vector analysis point clouds provide qualitative and quantitative representations of morphology. Regional dysmorphology in craniosynostosis cases is demonstrated graphically. **Conclusions:** Three-dimensional vector analysis generated accurate, reproducible, and comprehensive craniofacial morphometric data. 3DVA may be used for paired data analysis (eg, a single subject undergoing surgical correction), comparative group data analysis, and craniofacial data archiving. The technique can provide objective characterization of craniofacial morphology previously not possible.

The treatment of congenital and acquired craniofacial conditions requires an understanding of normal and abnormal cranial form. In these conditions, expert opinions vary as to diagnosis, severity assessment, and treatment. Regarding objective methods of shape analysis, traditional anthropometric indices are most commonly employed; yet, they fail to provide a satisfactory representation of complete 3-dimensional (3D) skull morphology, which is far more complex than can be represented by any single measure or the collective interpretation of several measures.

Scaphocephaly, associated with sagittal synostosis, provides the most poignant example of the problems inherent to traditional methodology for cranial shape analysis. Scaphocephaly is typified by a long narrow head in which the parietal prominences are absent and frontal and occipital protrusions are conspicuous.^1^ Corrective procedures for sagittal synostosis are designed to restore normal head shape. However, the objective standard for “normal” head shape, both for developing a diagnosis of cranial dysmorphology and assessing the efficacy of corrective surgery is defined in the current literature only by simplistic parameters. Craniofacial outcome analyses often focus on subjective impressions derived by the treating surgeon. Available objective methods tend to focus on the most prominent features associated with a given condition. In the case of sagittal synostosis, these are the narrow biparietal dimension and the elongated antero-posterior dimension. The ratio of these measurements defines the cranial index (CI). The means by which CI is derived has varied greatly among different authors.^2^–^9^ The cranial index has advantages including (1) evaluation of the most obvious features of dysmorphology; (2) ease of measurement from computed tomography (CT) data; (3) validation against direct caliper measurement; and (4) availability of normative data.^6^

Despite these advantages, the cranial index has shortcomings that call into question the utility of simple indices in general. First, CI takes into account only length and width, failing to characterize any other dysmorphology or correction thereof. To illustrate this point, a rectangle and an ellipse are entirely different, but could potentially share the same width-length ratios. Cases of sagittal synostosis demonstrate widely varying patterns of frontal bossing, occipital bossing, bitemporal narrowing, and vertex height deficiency. Recently, Schmelzer et al demonstrated the significant patterned variability among cases of sagittal synostosis.^10^ Second, CI is a relative index with no predefined reference origin (zero point); therefore, CI cannot distinguish regional differences such as frontal and occipital bossing.^11^ Third, the technique used for measuring CI varies widely, and has been modified since the early caliper studies. Modern CT and graphic computational programming systems offer a greater potential to bring objective assessment closer to the subjective impression of this (and other) condition(s).

Previously, we demonstrated the use of single-plane vector analysis (mid-sagittal vector analysis) in overcoming the shortcomings of traditional analytic techniques in sagittal synostosis (Fig 1). The goal of the current study is to demonstrate the more general utility of computed multivector analysis in craniofacial anthropometry using semi-automated 3-dimensional vector analysis (3DVA). Using a set of vectors emanating at prescribed angles from a single fixed point, 3DVA provides a finite, yet comprehensive, dataset of defined cranial surface points. These data may be used to provide dimensional analysis and to quantify point-to-point spatial relationships in any plane.

## MATERIALS AND METHODS

This study is presented in 2 parts. In part I (validation), measurements derived using 3DVA surface points were tested for accuracy and reproducibility. In part II (application), a normative, age- and gender-stratified database of craniofacial morphology was constructed using 3DVA. Test cases of craniosynostosis were compared against matched normative data to determine the specific nature and extent of dysmorphology, using a quantitative graphical output.

### Three-dimensional vector analysis

**Figure 2:**
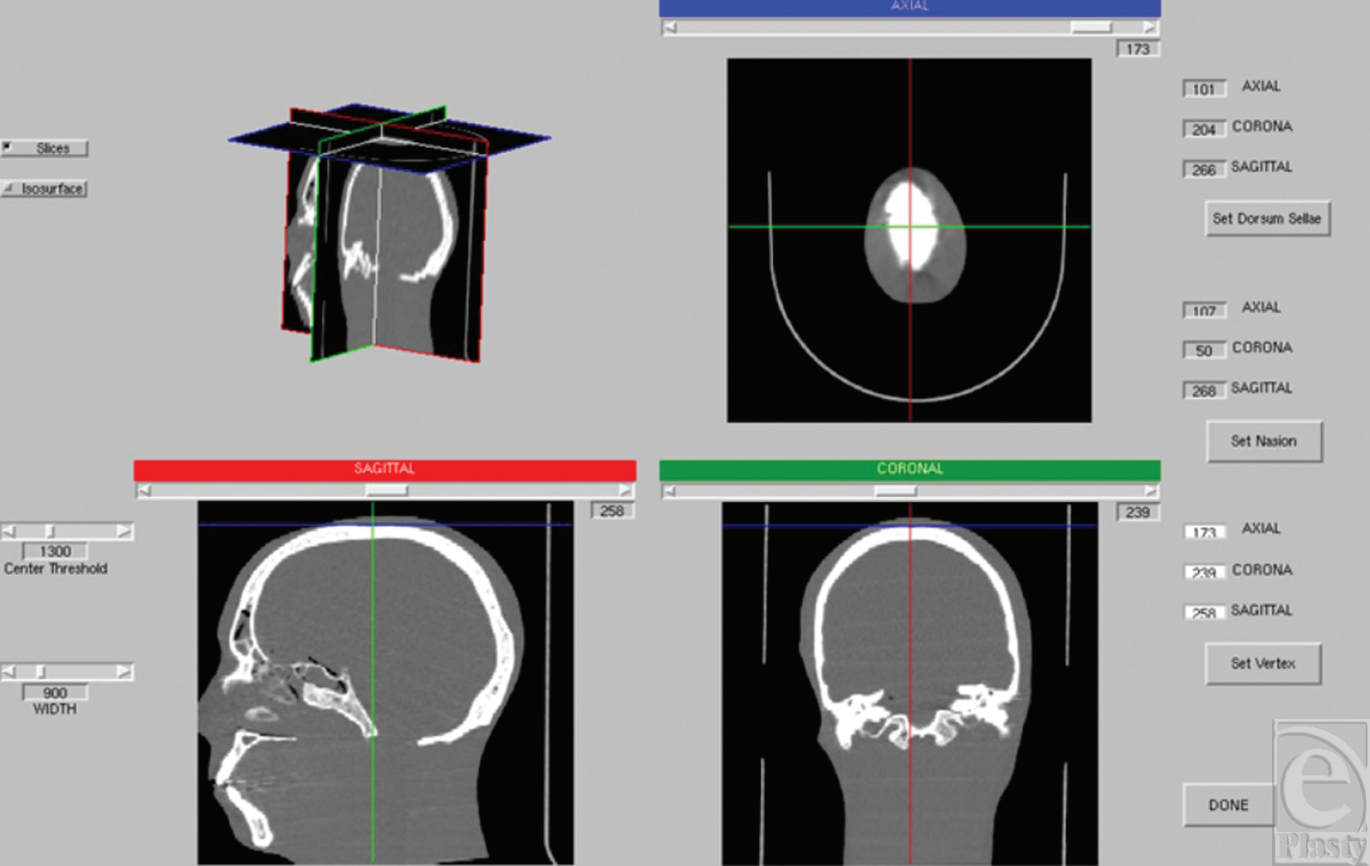
Orthographic viewer. After DICOM data are imported, the user defines the nasion, vertex, and dorsum sella in 3 planes viewed concurrently: axial, coronal, and sagittal. In this way, the analysis is corrected for rotation, translation, and alignment.

The 3DVA software was written using Matlab (R14) (The Mathworks, Natick, Mass) and was executed on a dual-core PowerMac G5. Raw Digital Imaging and Communications in Medicine data is imported into Matlab R14. Once the Matlab 3D matrix of the scan is generated, the reader is presented with an orthographic viewer showing concurrent axial, coronal, and sagittal views. The reader is prompted to select 3 points: dorsum sella, nasion, and vertex (Fig 2), which correct for translation, alignment, and rotation.

A bone threshold is defined, and the reader is prompted to enter a degree increment for the analysis. Vectors are generated regularly from the origin (dorsum sella) to the cranial surface at the specified degree increment.

**Figure 3:**
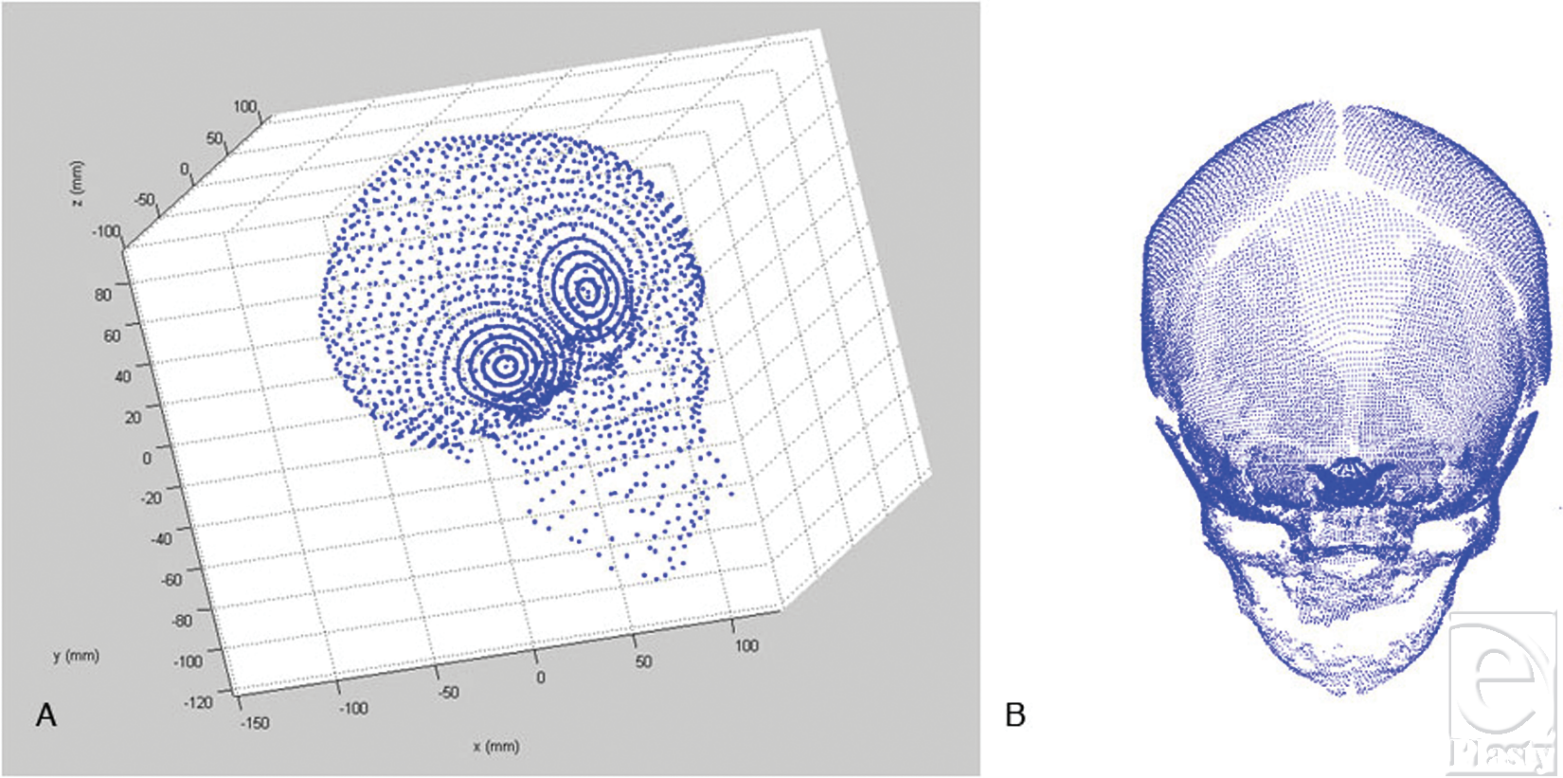
3DVA-generated cranial surface point clouds. (A) Automated measurement of vectors from dorsum sella to outer cortex of calvarium using 3DVA at a low-frequency interval of 10°. Subject is an adult human skull embedded in a doped-lucite matrix. (B) Automated measurement of vectors from dorsum sella to outer cortex of calvarium using 3DVA at a high-frequency interval of 2°. “Craniofacial Fingerprint.” Subject is a pediatric patient; open fontanelles are clearly discernable.

For each vector, the pixel furthest from the origin at the bone threshold is recorded as the cranial surface for that vector. Starting at the lowest elevation and azimuth, the vectors are generated sequentially for 360 degrees. Postanalysis, a 3D graphical representation of the points at the heads of the vectors is created (Fig 3), along with a tabular output of the azimuth, elevation, and magnitude associated with each vector. Some vectors generated for each skull will fail to define cranial surface points, and their magnitudes are recorded as “NaN” (not a number) in the output data tables. This occurs when a vector passes through a hole in the skull (ie, orbit or fontanelle) or misidentifies an image artifact as cranial surface.

**Figure 4:**
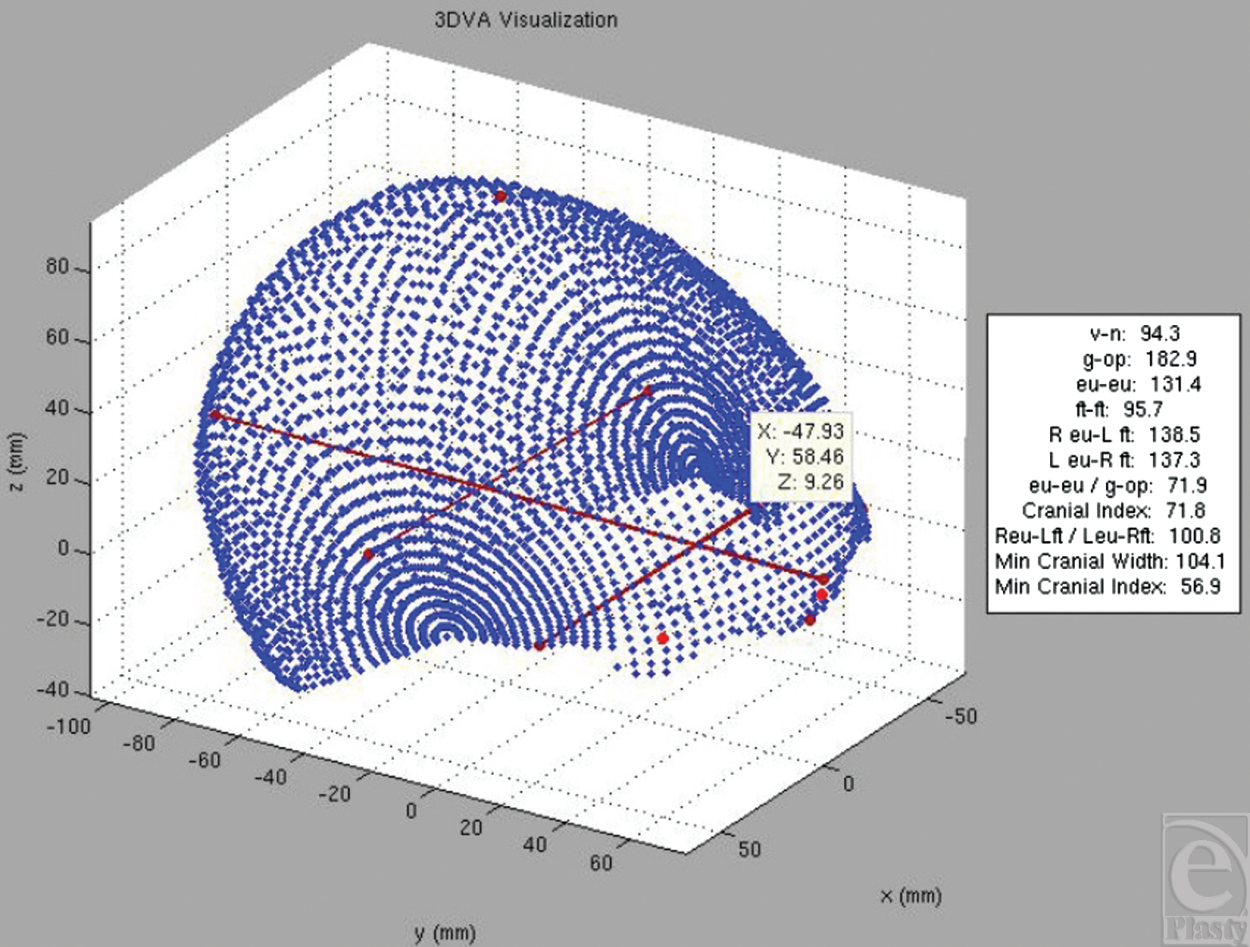
Output from 3DVA graphic viewer. Blue dots represent intersections of the vectors with the subject skull surface. A variety of traditional anthropometric landmarks and indices are selected/calculated automatically. The white box contains an automated output summary.

Any point can be selected on the graphic viewer, and any point-to-point relationship can be determined. The graphic representation (Fig 4) was programmed to use landmark identification to compute a set of traditional, clinically relevant anthropometric indices. Landmark selection can be done through 2 different methods: automated and manual point selection. 3DVA can automatically select certain landmarks defined by maximum or minimum values within a given angular range (such as the nasion, vertex, eurions, opisthocranion). More subjective or subtle landmarks can be manually defined by mouse click on a 3D graphical user interface. Hard-coded equations in the software calculate anthropometric indices using the Cartesian coordinates of the selected points. The 3DVA program was used to average multiple datasets together to create an age-incremental normative database and to perform comparative analyses between datasets. In 3DVA, if a single dataset is compared to an appropriate averaged control dataset, the graphic display demonstrates variance for each point in color-coded standard deviations from the control data.

## PART 1: VALIDATION

### Accuracy testing

A craniofacial phantom (Lucite embedded skull) was used to perform accuracy studies. Fiducial markers were placed on the phantom at 5 craniofacial anthropometric landmarks: vertex, right eurion, left eurion, nasion, and opisthocranion. The phantom dataset was processed on a Vitrea workstation (Vital Images, Minnetonka, Minn). CT-based anthropometric measurements were performed by placing the measurement tool on the cross points of fiducial markers and determining the intermarker distances. The Vitrea workstation, in routine clinical use, has been certified as accurate for evaluating distances and may be considered a criterion standard for measurements. For comparison, 3DVA was used to calculate intermarker distances between each of the 10 marker pairs. In 3DVA, each measurement was made 5 times.

For each test distance, the mean, standard deviation, and standard error of the mean for the five 3DVA trials were calculated. The means were then compared to the CT-based measurements and the absolute percentage deviations were determined.

### Reproducibility

Three trained readers independently used 3DVA to evaluate the phantom dataset and 5 normative pediatric head CT datasets (boys aged 13, 13, and 81 months; girls, 14 and 19 months). Each reader analyzed each dataset 5 times. The evaluations were performed using 10° sampling increments, which generated 437 cranial surface points to be analyzed for each dataset.

Reproducibility of data was examined via statistical analysis of both intraobserver and interobserver precisions. For all analyses, the skull phantom and the set of pediatric scans were analyzed separately.

#### Intraobserver analysis

Data were averaged across the 5 trials that a given reader ran on a given skull, to calculate the mean and standard deviation of the magnitude for each of the four hundred thirty-seven 3DVA-generated cranial surface-defining vectors. Thus, an average representation of each skull was determined for each user. For each elevation/azimuth pair, if any of the user's trials resulted in a magnitude recorded as NaN, the mean and standard deviation for that vector were also recorded as NaN, and excluded from further analyses. The remaining standard deviations were averaged over the entire skull to generate a skull dataset standard deviation for each user. Similarly, the means were averaged to generate a skull dataset mean magnitude. To reduce these data to a single descriptive statistic for the entire set of pediatric scans across all users, the 15 dataset standard deviations (5 dataset standard deviations for each of 3 users) were averaged together to generate the “mean standard deviation” for each skull (the mean standard deviation for the skull phantom was simply the dataset standard deviation calculated for it). The “mean magnitude” and “mean standard error of the mean” were calculated in the same way. The coefficient of variation for each skull was also calculated as the mean standard deviation divided by the mean magnitude. Finally, the mean intraobserver error estimate was calculated as the mean of the standard deviation of the natural logs of the vector magnitudes.

#### Interobserver analysis

For each skull and each pairing of users, the difference in vector magnitude at each (definable) point on the users' average representations of the skull surface was calculated. The absolute values of the differences were averaged across the skull to create a mean magnitude difference. The 15 mean magnitude differences (1 for each of 3 possible user pairings for each of the 5 pediatric skulls) were averaged to generate a single statistic to describe interuser variability (the variability for the phantom was simply the mean of the differences from the 3 user pairings).

## PART II: APPLICATION

### Normative database

Standard CT datasets from pediatric patients were obtained under the auspices of the Duke University Institutional Review Board. Studies for the normative database were retrospectively acquired and deidentified for the following ages: 1, 3, 6, 9, 12, 18, 24, 26, 48, and 60 months. Studies with movement artifact or incomplete visualization of the cranium were excluded. The scans used in the normative database had been performed for a variety of clinical indications but were determined to be “normal” by the attending radiologist. Datasets were processed using 3DVA software and entered into the database (Table 1).

### Craniosynostosis

Preoperative Craniofacial CT datasets from pediatric patients with craniosynostosis were obtained under the auspices of the Duke University Institutional Review Board. The CT studies were retrospectively acquired and deidentified as described above and were processed in 3DVA. Each case was paired to the sex-/age-matched, averaged dataset in the normative database.

## RESULTS

### Accuracy

Three-dimensional vector analysis was found to be highly accurate. All standard errors of the mean for 3DVA-derived intermarker measurements were less than 0.5 mm (Table 2). The absolute percentage differences between 3DVA-derived measurements and CT-based measurements were less than 1% with a mean of 0.52% (Table 3).

### Reproducibility

Indefinable points resulted from all pediatric skull analyses, leaving an average of 424.6 points to be analyzed for each skull. Both the mean standard deviation and the mean standard error are less than 0.5 mm. All errors and variabilities (for both interobserver and intraobserver analyses) are less than 1%. The errors are greater for the pediatric head CT than the phantom craniofacial CT data, 0.67%/0.91% versus 0.45%/0.51%, respectively. Furthermore, interobserver error is greater than intraobserver error for both types of data (0.51%/0.91% vs 0.45%/0.67%). The *t*- test performed on the 6 statistical measures yielded a *P* = .18, meaning that the 2 types of datasets do not result in statistically significantly different results (Table 4).

### Dysmorphology versus normative representations

**Figure 5:**
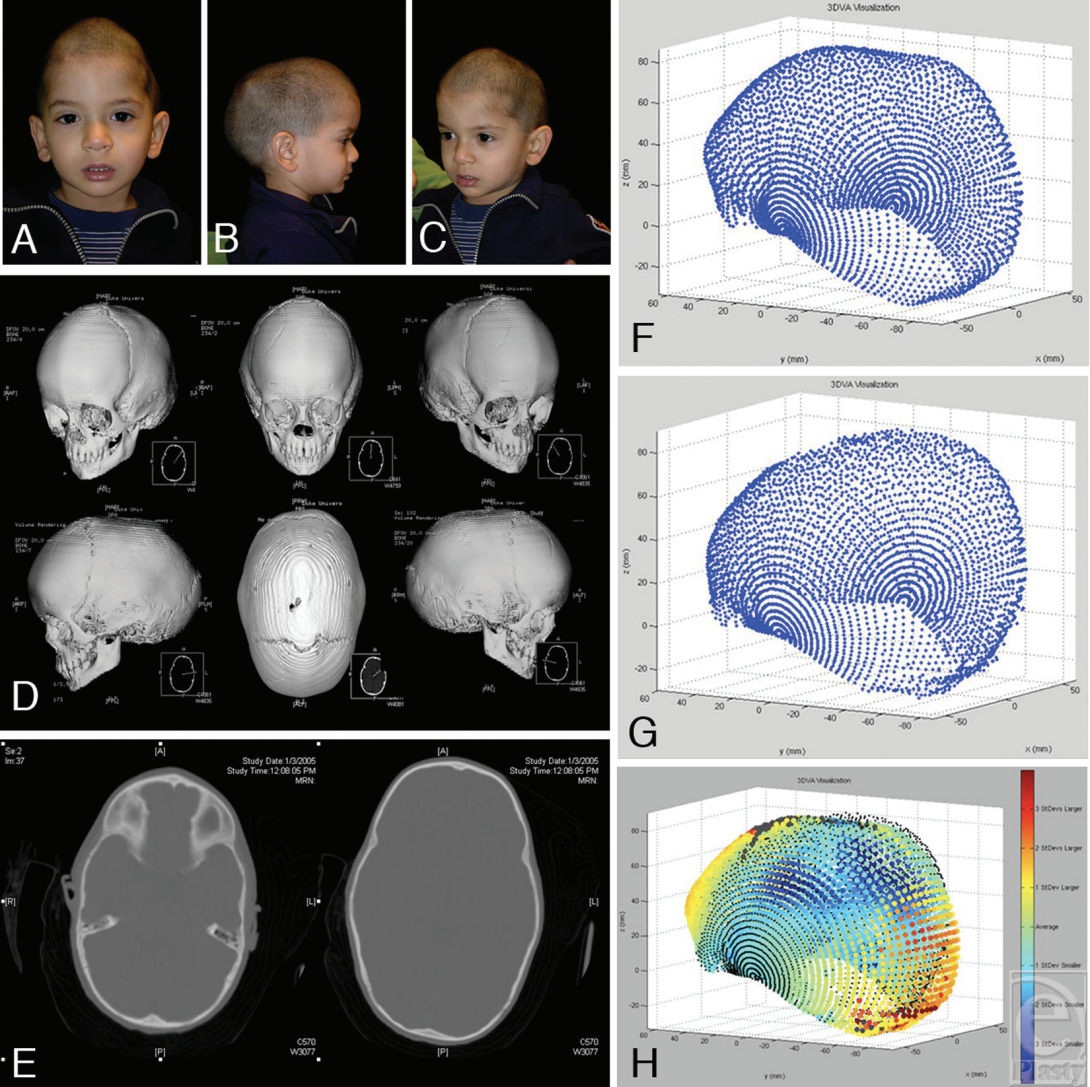
Sagittal synostosis. (A, B, and C) Nine-month-old with narrow bitemporal width, AP elongation, high anterior vertex, but no frontal bossing. (D and E) 3D CT demonstrating sagittal synostosis. The closed metopic suture perhaps contributed to this particular shape pattern, atypical for scaphocephaly. (F) 3DVA-generated point cloud for the patient's DICOM data. (G) 3DVA-averaged data for normal 9-month-old males (*N* = 4). (H) Subject's data set (colored points) compared with normative data set (black points). The color scale describes each point's distance (in number of standard deviations) from the population mean. Note anterior vertex and low occipital bossing and high occiput recession.

**Figure 6:**
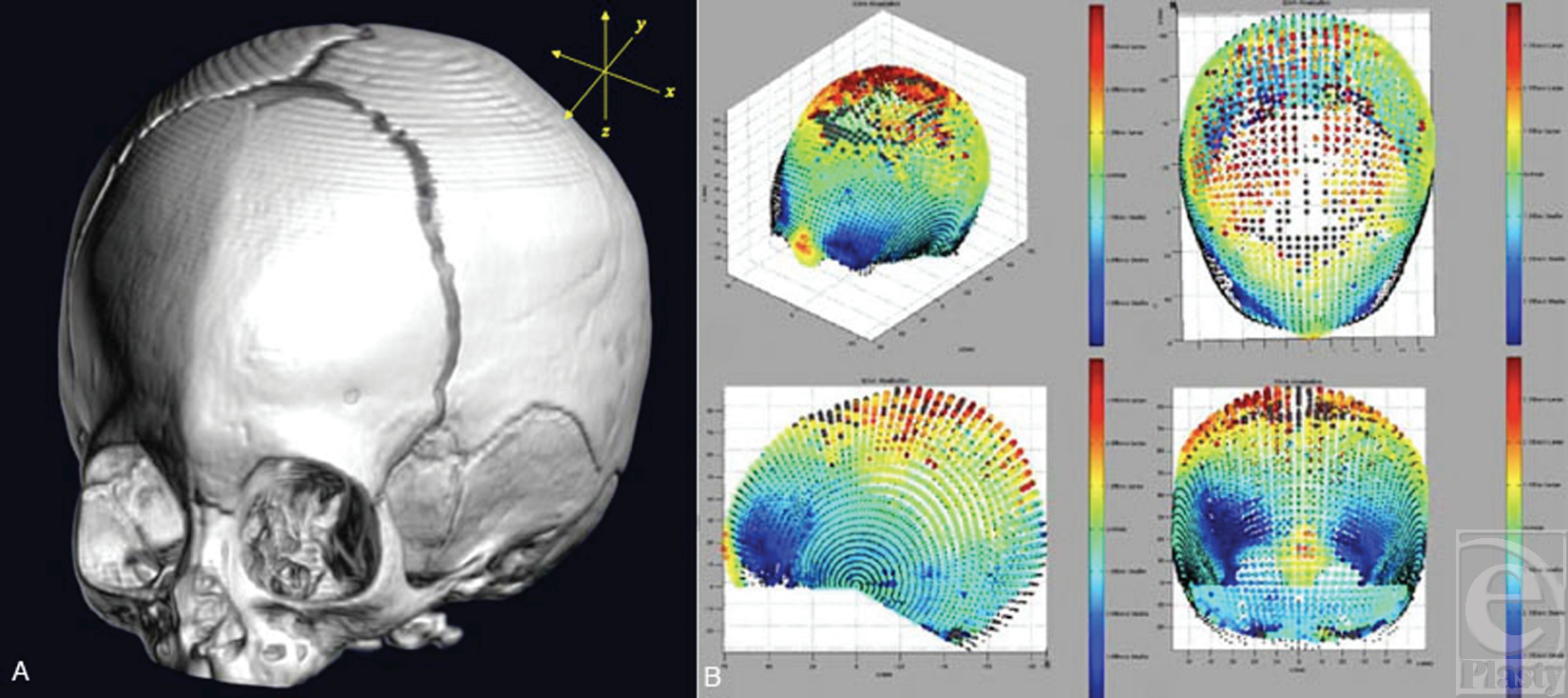
(A and B) 3DVA is applied to a subject with metopic synostosis. The subject data are again pared with an averaged, age/gender-matched, normative data set. The characteristics of tringonencephaly are easily appreciable and are quantified in 3DVA based on standard deviations from the norm.

The 3DVA-created point clouds created for craniosynostosis cases were plotted against age- and gender-matched normative datasets to generate color-coded point clouds (Figs 5 and 6). The color scale corresponds to the difference in vector magnitude between the craniosynostotic and normative skulls in terms of standard deviation from normal. Each case demonstrates distinct differences.

## DISCUSSION

Consensus has not been reached in the treatment of craniosynostosis, particularly for sagittal synostosis. Disparity in opinion is explained at least in part by the limited evidence available to support the superiority of any one approach over another. In order to substantiate the merits of any given technique, objective, serial quantitative data are required. Objective analytic methods must be able to demonstrate both the variation from normal among affected individuals and the longitudinal change for individuals following surgery.

The CI is the most commonly referenced measure. The ease of obtaining data and the availability of normative data are significant advantages of CI. In early documentation of normative head shape, caliper measurements were used to directly obtain data.^12^–^15^ Later, the CI was determined from measurements derived from anterior projection and lateral cephalometric radiographs.^2^–^4^,^7^ Currently, most investigators employ CT imaging for this purpose. Waitzman et al^16^,^17^ have demonstrated the accuracy of measurements obtained using axial CT images compared with those derived from direct caliper measurements of phantoms (dry skulls).

As a single parameter, the CI does not confer understanding of regional severity or specificity. One may gather additional indices to create a larger parameter set, but the collective interpretation of such a list is difficult to apply in the clinical setting. Some have concluded that quantitative measurements simply confirm clinical observations, stating in deference to the cranial index that, “…no methods have been described to quantify or objectively measure frontal bossing, midline ridging, and occipital protuberance…”11 (p146)

Three-dimensional vector analysis was developed to address inadequacies of current cranial anthropometric techniques. It captures the significant findings that differentiate diagnoses, and it captures the subtle variations of individuals with the same diagnosis.

Three-dimensional vector analysis is highly accurate and reproducible; it allows archiving of its output data in tabular form, which enables data averaging—particularly useful in academic endeavors. Furthermore, the graphic interface allows for easy clinical interpretation via the intuitive 3D display. Applied to preoperative CT data, it would enable automated analysis of diagnosis and severity. Such analyses, in theory, could be incorporated into a clinical postprocessing radiology workstation. Preoperative- and postoperative datasets could be paired for outcome analysis. Each dataset could be compared independently to the normative dataset, or the 2 could be compared to one another, with the regional differences expressed in millimeters of absolute change along each vector.

It is important to consider other evolving technologies in the field of morphometric analysis. One noteworthy example is 3D photogrammetry, the role of which in cranial dysmorphology analysis has yet to be determined. The inability of this modality to obtain an internal reference lends it to limitation in standardization, validation, and coregistration for paired analysis or for normative comparison. However, an advantage of this technique is that it does not require radiation exposure.

Radiation dose reduction in CT has become an important initiative in medicine. Past work has shown that a decrease in tube voltage from 140kVp to 100kVp reduces organ and effective doses by approximately two thirds. In addition, a linear decrease in dose occurs by reducing tube current, such that dose reduction of up to 90% can be obtained over standard protocols. At the authors' institution, where routine CT for preoperative and postoperative evaluation are performed, dose reduction has become a priority. Three-dimensional photogrammetric techniques may prove more valuable in the analysis of conditions that would not otherwise require evaluation by CT (ie, positional molding). However, in most craniofacial centers, some form of head CT is generally performed preoperatively for cases of craniosynostosis to confirm the diagnosis and for evaluation of the brain.

## STUDY LIMITATIONS

One difficulty encountered in building a normative database is the acquisition of sufficient retrospective data. It would be unethical to subject a child to needless radiation, so scan data must be collected from children for whom CT scans are clinically indicated. Furthermore, only scans that are free of any artifact or motion and demonstrate no abnormality can be used to build the database. Normative scans for the younger age, important for preoperative assessment of craniosynostosis, are most difficult to acquire. To help overcome this limitation, the database is being expanded and new scans are being entered continuously.

Another potential limitation of the 3DVA algorithm is the current coregistration technique. While using the dorsum sella, nasion, and vertex to align crania is sufficient grossly, this method permits systematic, user, and random error. Despite 3-point coregistration, other craniofacial landmarks may not be in positions that minimize the displacement for the entire cranium. A related point of systematic error is that if the coregistration is inadequate, then a given angular pair will not map onto its corresponding angular pair in another dataset, thereby skewing the 3DVA analysis.

Finally, the methods assume the dorsum sella to be a stable point. If the location of the dorsum sella, which is set as the origin, varies from one subject to another, then again you have the type of systematic error, which results in inappropriate vector comparisons. The literature suggests that cranial base variation (which leads to variation in dorsum sella location) can occur in some forms of craniosynostosis. However, in relatively symmetric forms of craniosynostosis, resulting from premature fusion of a midline suture (ie, sagittal or metopic), a shift of the origin point is less of a concern than it is for asymmetric dysmorphologies, such as those encountered in unilateral coronal synostosis.

## CONCLUSIONS

Substantiation of a surgical technique to provide normalization of head shape for any form of dysmorphology requires objective analytic methodology. Shortcomings of simple indices, like CI for sagittal synostosis, are discussed herein. Mid-sagittal vector analysis was developed originally to address issues related to scaphocephaly. Three-dimensional vector analysis provides quantitative and graphical representation of craniofacial morphology—in general. More broadly applied, it allows creation of a digital “craniofacial fingerprint.” From this craniofacial fingerprint, an unlimited range of assessment tools may be derived. Three-dimensional vector analysis may be used for the purpose of paired data analysis (as in a single subject undergoing surgical correction), comparative group data analysis through averaging, and craniofacial data archiving. The technical requirements are well within the capabilities of most centers performing advanced neuroimaging and craniofacial surgery. Application to a wide range of conditions may be possible; however, for such comprehensive application, this instrument requires further refinement.

## Acknowledgments

This work was supported by research grants from the Southeastern Society of Plastic and Reconstructive Surgeons, Stryker-Leibinger Corporation, and the General Electric Corporation.

## Figures and Tables

**Figure 1:**
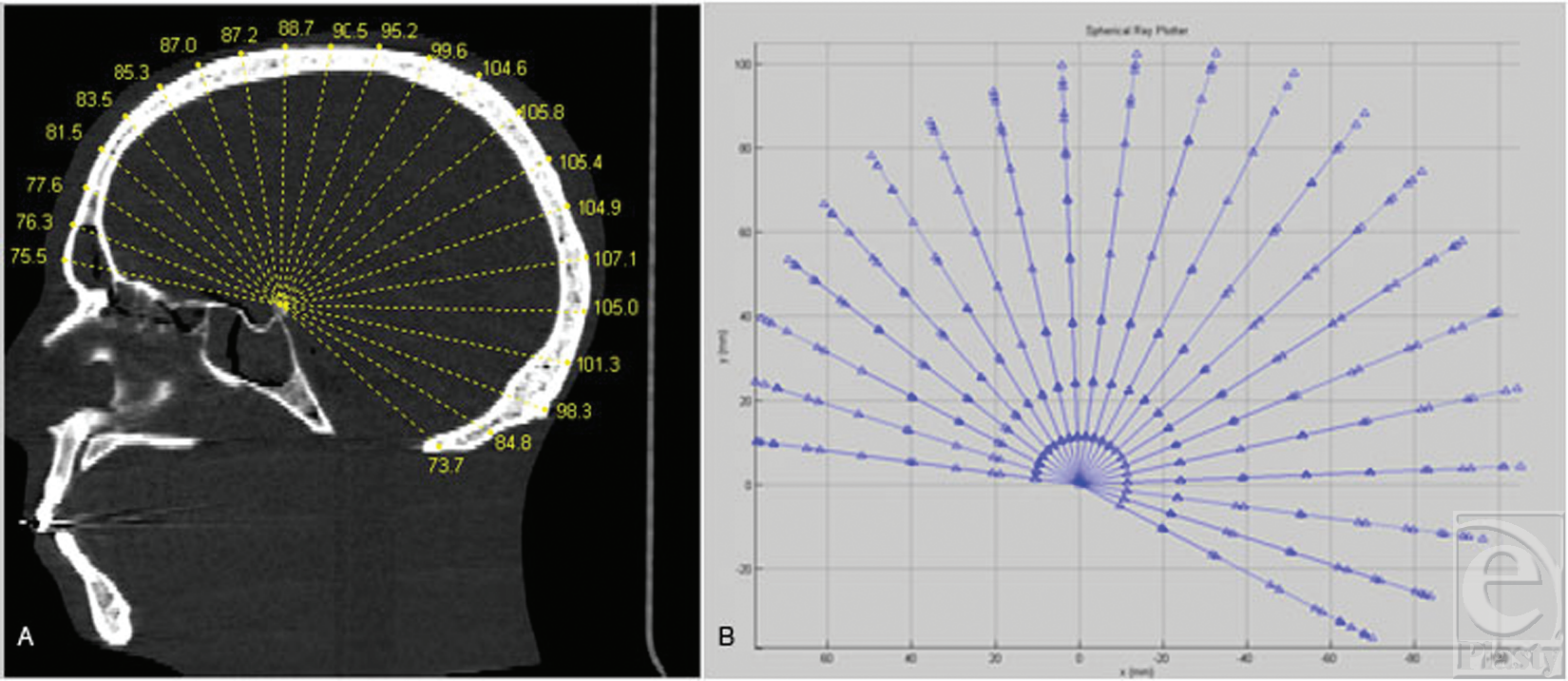
The mid-sagittal ray analysis, precursor to 3DVA. (A and B) Analysis is performed in the mid-sagittal plane only. A set of radial vectors at 10° increments eminates from the posterior ledge of the sella. Measurements are taken to the outer table. This analysis localizes regional differences (like frontal vs occipital bossing in scaphocephaly) but is limited to 2 dimensions.

**Table 1:** Normative database inventory

Age, mo	Male	Female	Total
1	2	1	
3	2	2	
6	2	2	
9	5	2	
12	2	2	
18	5	2	
24	1	6	
36	2	3	
48	8	1	
60	6	5	
Total	35	26	61

**Table 2:** Accuracy study—three-dimensional vector analysis measurements*

Intermarker measurement	Trial 1, mm	Trial 2, mm	Trial 3, mm	Trial 4, mm	Trial 5, mm	Mean, mm	SD, mm	SE, mm
v-n	124.25	123.72	124.29	123.7	124	124	0.27	0.12
n-op	196.79	197.24	197.7	197.3	196.4	197.07	0.52	0.23
n-L eu	99.34	98.69	98.36	98.51	98.03	98.59	0.49	0.22
n-R eu	95.92	94.98	95.02	94.72	94.72	95.07	0.49	0.22
v-op	128.46	128.73	129.3	129.8	128.5	128.94	0.57	0.25
v-L eu	110.31	108.75	110.65	109.1	110.7	109.9	0.9	0.4
v-R eu	105.19	104.49	105.89	105.8	105.9	105.46	0.62	0.28
op-L eu	146.41	146.94	147.72	148.4	147.2	147.32	0.75	0.33
op-R eu	147.45	146.93	147.95	147.6	146.9	147.36	0.44	0.2
L eu-R eu	140.49	138.51	138.98	139.4	139	139.28	0.75	0.34

*v indicates vertex; R/eu, right eurion; L/eu, left eurion; n, nasion; and op, opisthocranion.

**Table 3:** Accuracy study—3DVA measurement comparisons to CT-based measurements.*

Intermarker measurement	CT-based measurement mm	Mean 3DVA measurement mm	Absolute deviation
v-n	124.7	124	0.56
n-op	199	197.07	0.97
n-L eu	98.4	98.59	0.19
n-R eu	95.6	95.07	0.55
v-op	128.8	128.94	0.11
v-L eu	110.1	109.9	0.19
v-R eu	104.6	105.46	0.82
op-L eu	148.8	147.32	0.99
op-R eu	148.6	147.36	0.83
L eu-R eu	139.3	139.28	0.01
Mean absolute deviation			0.52

*3DVA indicates three-dimensional vector analysis; CT, computed tomography; v, vertex; R/eu, right eurion; L/eu, left eurion; n, nasion; and op, opisthocranion.

**Table 4:** Reproducibility study—comparison of craniofacial CT analysis with head CT* analysis

	Phantom craniofacial CT	Pediatric head CT
Mean number of indefinable points	0	12.4
Median number of indefinable points	0	5
Mean standard deviation, mm	0.34	0.48
Mean standard error, mm	0.02	0.02
Coefficient of variation, %	0.45	0.67
Mean intraobserver error estimate, %	0.45	0.68
Mean interobserver variability, mm	0.39	0.64
Mean interobserver variability as a percentage of mean magnitude, mm	0.51	0.91

*CT indicates computed tomography.
